# Comparison Study between ERCP and PTBD for Recurrent Choledocholithiasis in Patients Following Gastrectomy

**DOI:** 10.3390/diagnostics13162651

**Published:** 2023-08-11

**Authors:** O Seong Kweon, Jun Heo, Min Kyu Jung

**Affiliations:** 1 School of Medicine, Kyungpook National University, Daegu 41944, Republic of Korea; 2 Department of Internal Medicine, Kyungpook National University Hospital, Daegu 41944, Republic of Korea

**Keywords:** gastrectomy, choledocholithiasis, endoscopic retrograde cholangiopancreatography, percutaneous transhepatic biliary drainage, recurrence

## Abstract

The recurrence rate of choledocholithiasis in the general population has been reported to exceed 10%. The incidence of cholelithiasis was reported to be higher in patients following gastrectomy than that in the general population. However, there is no study for recurrent choledocholithiasis incidence in patients following gastrectomy. This study aimed to evaluate the recurrence rate of choledocholithiasis and identify risk factors for recurrent choledocholithiasis in patients following gastrectomy. A retrospective analysis was performed on patients with gastrectomy history who underwent choledocholithiasis removal in Kyungpook National University Hospital between January 2011 and December 2019. Choledocholithiases were treated by endoscopic retrograde cholangiopancreatography (ERCP) (*n* = 41) or percutaneous transhepatic biliary drainage (PTBD) (*n* = 90). The gastrectomy type was classified as subtotal gastrectomy with Billroth I (18.3%), Billroth II (45.0%), and total gastrectomy with Roux-en-Y (36.6%). During a median follow-up period of 31.5 (range, 6–105) months, choledocholithiasis recurrence was noted in 19 of 131 patients (14.5%). In subgroup analysis, the ERCP group (24.4%) had higher choledocholithiasis recurrence than the PTBD group (10.0%). Stone removal modality (ERCP), no use of balloon sphincteroplasty, and the presence of periampullary diverticulum were significant risk factors for recurrent choledocholithiasis. In multivariate analysis, ERCP (hazard ratio (HR), 3.597; 95% confidence interval (CI): 1.264–10.204) CBD stricture (HR, 3.823; 95% CI: 1.118–13.080) and no use of balloon sphincteroplasty (HR, 4.830; 95% CI: 1.669–13.889) were risk factors for recurrent choledocholithiasis following stone removal. The incidence of CBD stones in patients who underwent gastrectomy is similar to that of the general population. ERCP, CBD stricture, and no use of balloon sphincteroplasty are potential risk factors for recurrent CBD stones following gastrectomy. When we consider PTBD disadvantages, the ERCP procedure with active use of balloon sphincteroplasty is recommended to decrease recurrent CBD stones.

## 1. Introduction

The incidence of choledocholithiasis is approximately 10–15% in the general population [1]. Choledocholithiasis exhibits a wide range of clinical manifestations, with most cases being symptomatic. Right upper quadrant pain, nausea, vomiting, fever, and jaundice are the common symptoms. If cholangitis due to common bile duct (CBD) stones is not properly treated with antibiotics and timely intervention, including endoscopic retrograde cholangiopancreatography (ERCP) or percutaneous transhepatic biliary drainage (PTBD), it can lead to sepsis and, in serious cases, even death [2,3].

Several recent studies have reported that gallbladder (GB) stone incidence increased following gastrectomy [4]. Researchers have hypothesized that gastric hypomotility, which occurs following surgery, disrupts bile flow in the GB, thereby leading to bile stasis [5]. In the same context, one study has suggested that concomitant cholecystectomy could be beneficial in preventing cholecystitis, a condition that is highly likely to develop following gastrectomy [5,6,7]. However, CBD stone formation after gastrectomy is not well established.

Furthermore, the altered anatomy resulting from gastrectomy may pose challenges for the ERCP procedure, particularly in cases of total gastrectomy. A short-type balloon enteroscope is currently available for ERCP in the Roux-en-Y reconstruction anatomy. However, the long Roux and pancreatobiliary surgical limbs are still hurdles for advancing the scope into the target site with minimal bowel loop formations [8]. The difficulty of the procedure due to structural changes can ultimately lead to incomplete bile duct stone removal, subsequently resulting in a high recurrence rate.

However, to date, no other study has validated the occurrence of CBD stone recurrence following gastrectomy. Therefore, we analyzed the CBD stone recurrence rates in patients with gastrectomy history. Additionally, we compared the recurrence rates on the basis of treatment modalities (ERCP or PTBD) and identified risk factors contributing to an increased CBD stone recurrence rate in patients with a history of gastrectomy.

## 2. Materials and Methods

### 2.1. Patients

A retrospective analysis was performed on patients who underwent CBD stone removal via ERCP or PTBD between January 2011 and December 2019 for choledocholithiasis following gastrectomy.

The following were the inclusion criteria: (1) eligible patients underwent first-time CBD stone removal via ERCP or PTBD and (2) a history of gastrectomy before CBD stone removal. The following were the exclusion criteria: (1) with a history of prior CBD stone removal via ERCP or PTBD; (2) a history of gastrectomy following CBD stone removal; (3) no definite presence of CBD stone or sludge (e.g., suspicious sphincter of Oddi dyskinesia and passed CBD stone); (4) follow-up duration < 1 year following CBD stone removal; (5) PTBD not removed; and (6) biliary malignancy directly influencing the CBD stone (e.g., GB, bile duct, ampulla of Vater, and pancreatic cancers); (7) history or case of surgical CBD stone removal (e.g., exploration of the CBD). The study design was reviewed and approved by the Institutional Review Board of Kyungpook National University Hospital, Republic of Korea (approval no. 2023-02-015-001). The study protocol conforms to the ethical guidelines of the 1975 Declaration of Helsinki as reflected in a prior approval by the institution’s Human Research Committee.

### 2.2. ERCP Method

Two experienced gastrointestinal endoscopists (Professors J.M.K. and H.J.) performed the ERCP procedures in this study period. A duodenoscope (TJF 260V or JF 260V, Olympus, Tokyo, Japan) was used with fluoroscopic guidance in the ERCP rooms. Two or three nurses assisted with the procedures. The endoscopists used papillotomes using the guidewire technique in almost all ERCP procedures. Balloon sphincteroplasty is a procedure that uses a balloon catheter for relieving blockages or strictures in the sphincter of Oddi, which can lead to problems with the flow of bile and pancreatic fluid. The endoscopists performed the balloon sphincteroplasty procedure and balloon size selection. Following CBD stone confirmation using fluoroscopy, a basket or retrieval balloon catheter was used as per endoscopists’ discretion. Furthermore, endoscopic papillary large balloon dilatation or endoscopic retrograde biliary drainage stent insertion was performed by the endoscopists. Endoscopic retrograde pancreatic duct drainage with a stent was also performed in cases with a high possibility of pancreatitis following ERCP. Saline irrigation (40–200 cc) was sometimes used for the removal of suspicious remnant CBD stones or sludge into the duodenum with a retrieval balloon catheter.

### 2.3. PTBD Method

Two experienced interventional radiologists (L.S.Y. and C.J.G) performed PTBD. Under sonographic guidance and fluoroscopic assistance, the puncture was performed using a 21-gauge Chiba needle (Cook, Bloomington, IN, USA) on the transhepatic approach. Subsequently, 0.035-inch guidewires (Terumo, Tokyo, Japan) were typically inserted through the needle, and an 8.5-French drainage catheter (Cook) was inserted into the intrahepatic duct. Stone removal was typically performed the day after PTBD insertion. A basket (Cook) was usually used. Following CBD stone removal, balloon sphincteroplasty was performed as per the interventional radiologists’ discretion. Moreover, saline irrigation was used for the removal of suspicious remnant CBD stones into the duodenum.

### 2.4. Follow-Up

Following CBD stone removal, patients were usually treated for 2–3 days in the hospital. After discharge, they were followed up in the outpatient department every 4–12 weeks until 1–2 years. If they did not visit the outpatient department after 6 months, we called them to inquire regarding symptoms or any treatment in other hospitals.

### 2.5. Definition of CBD Stone Recurrence

A recurrent CBD stone is considered if the CBD stone is visible in abdominal imaging, including ultrasonography, computed tomography, or identified on cholangiogram during an ERCP procedure.

### 2.6. Study Outcomes

The primary outcome was CBD stone recurrence rate. Secondary outcome was identifying factors that influence CBD stone recurrence.

### 2.7. Statistical Analysis

All statistical analyses were performed using Statistical Package for the Social Sciences (version 22, IBM, Chicago, IL, USA). Statistical analyses of the results were performed using the chi-square test. Fisher’s exact test and Student’s *t*-test were used for categorical and normally distributed data, respectively. Recurrence was analyzed using the Cox proportional hazard model test as a survival model. For all analyses, *p*-values < 0.05 were considered statistically significant.

## 3. Results

### 3.1. Baseline Characteristics

**Figure 1:**
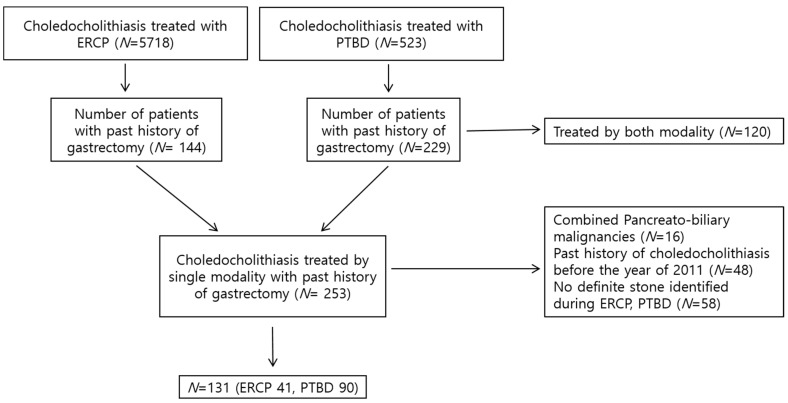
Flow chart of participants through study.

The baseline characteristics of enrolled patients in this study are shown in Table 1. Of the 373 patients with CBD stones following gastrectomy who underwent treatment with either ERCP or PTBD, 242 were excluded for not meeting the inclusion criteria, thereby leaving 131 included in the final analysis (Figure 1). Of the 131 patients, 76.3% (*n* = 100) were male participants. The mean age was 72.0 ± 9.4 (standard deviation (SD)) years. The Charlson comorbidity index score was 3.4 ± 1.9 (mean ± SD) points. Of the 131 patients, 91 (68.7%) underwent treatment with PTBD, whereas 41 (31.3%) received treatment with ERCP. Regarding the gastrectomy type, patients predominantly underwent subtotal gastrectomy with Billroth II anastomosis (*n* = 59, 45.0%), followed by total gastrectomy (*n* = 48, 36.6%) and subtotal gastrectomy with Billroth l anastomosis (*n* = 24, 18.3%). The characteristics of CBD stones could only be identified in patients who underwent ERCP. Among the identified CBD stone types, the brown pigment stone was observed to be the most predominant (28 of 31 identified stones, 90.3%). The mean CBD stone size was 9.5 ± 4.4 (SD) mm.

### 3.2. Recurrence Rate

The subgroup characteristics of the 131 participants divided into the recurrent and non-recurrent groups are presented in Table 2. No significant differences were noted in age, sex, history of cholecystectomy, and GB stones/sludge between the two groups. During a median follow-up period of 31.5 (range, 6–105) months, choledocholithiasis recurrence was noted in 19 of 131 patients (14.5%). Based on the comparison between the two groups, stone removal modality (ERCP, *p* = 0.030), no use of balloon sphincteroplasty (*p* = 0.004), and the presence of periampullary diverticulum (*p* = 0.039) were significant risk factors for recurrent choledocholithiasis. 

The subgroup characteristics of patients based on their treatment modality, with 41 and 90 patients undergoing ERCP and PTBD, respectively, are shown in Table 3. No significant differences were observed in terms of age, sex, or Charlson comorbidity index scores between the two groups. In the ERCP group, the patients were nearly evenly distributed to subtotal gastrectomy with Billroth I and II (48.8% and 51.2%). However, total gastrectomy patients were only treated by PTBD (53.3%). The CBD stone recurrence rate in patients treated with ERCP was 24.4%, which was not significantly higher (*p* = 0.057) than the 10.0% noted in the PTBD-treated group. The use of Balloon sphincteroplasty was higher in the PTBD group than in the ERCP group (75.6% vs. 58.5%, *p* = 0.001). Moreover, patients in the PTBD group tended to utilize larger balloons for sphincteroplasty than those in the ERCP group. The proportion of patients using balloons larger than 12 mm was 58.9% in the PTBD group, which was significantly higher (*p* = 0.001) than the 26.8% observed in the ERCP group. The number of procedures needed for CBD stone removal was different between PTBD and ERCP groups (3.71 ± 1.23 vs. 1.39 ± 0.67 (mean ± SD) times, *p* < 0.001).

The results of the multivariate analysis of risk factors and recurrent choledocholithiasis are shown in Table 4. In multivariate analysis, ERCP (hazard ratio (HR), 3.597; 95% confidence interval (CI): 1.264–10.204), CBD stricture (HR, 3.823; 95% CI: 1.118–13.080) and no use of balloon sphincteroplasty (HR, 4.830; 95% CI: 1.669–13.889) were risk factors for recurrent choledocholithiasis following stone removal.

## 4. Discussion

The incidence of gallstones following gastrectomy is 3.6% to 25.0% in Asia. However, it is nearly impossible to know the exact incidence of gallstones after gastrectomy because most gallstone patients are asymptomatic [5,9,10]. The incidence of gallstones tends to increase with time after gastrectomy. The incidence of gallstones was reported as a 5-year incidence of 13.6% and a 10-year of 22.1% [9]. In the general population, the prevalence of gallstones increases according to age. In 60-year-olds, the prevalence is reported as 7.3–7.9% in males and 8.2–11.9% in females in the United States and China [11,12]. Therefore, patients who underwent gastrectomy should be informed of the higher risk of gallstone formation. Abdominal imaging of ultrasonography or computed tomography is needed for gastrectomy patients with alarm symptoms such as right upper quadrant abdominal pain or dyspepsia.

The hypothesis that gastrectomy leads to a higher GB stone incidence is based on cholestasis in the GB, which can cause cholesterol formation. An increase in the cholesterol ratio contributes to cholesterol stone formation in the GB. During gastrectomy, vagal nerve branch injury or damage can occur. This result may reduce GB contractions. Another aspect is an alteration in gastrointestinal anatomy. The passage time of food material in the duodenum may be increased in patients after gastrectomy. This change decreases cholecystokinin secretion, which influences GB function [4,10]. The types of gastrectomy can also influence the incidence of gallstone formation. A larger extent of gastrectomy has a higher risk of damage to surrounding blood vessels, vagal nerves, and other tissues. In a cohort study of 698 gastrectomy patients, the incidence of gallstones was significantly higher in total gastrectomy and proximal gastrectomy than in distal gastrectomy (38.5% and 6.2% vs. 1.0%, *p* = 0.000) [4]. Among the subtotal gastrectomies, the Billroth II operation showed a higher incidence of gallstone formation than the Billroth Ⅰoperation. Although the mechanism was not fully understood, the change in the pattern of cholecystokinin secretion according to the type of subtotal gastrectomy might have a role in gallstone formation [9].

Based on this theory, it was believed that CBD stones developed in patients with a gastrectomy history were predominantly cholesterol stones. However, the most common type was brown pigment stones among the stones that were identified in the ERCP group in this study. Additionally, our study showed that cholecystectomy did not decrease CBD stone recurrence. The brown-pigmented CBD stones are primary CBD stones. This finding could be the main reason that cholecystectomy did not decrease the CBD stone recurrence. A recent study also concluded that a routine prophylactic cholecystectomy is not recommended after gastrectomy. During median follow-up of 48 months in 1480 patients after gastrectomy, 106 patients (7.2%) had developed gallstone formation, and only 9 patients had gallstone-related symptoms. CBD stones occurred in 20 patients (1.4%). Therefore, prophylactic cholecystectomy is not essential during gastrectomy. Other risk factors such as age, sex, body mass index, and diabetes also contribute to gallstone formation. These factors should be considered for the decision of prophylactic cholecystectomy at the time of gastrectomy [13].

The CBD stone recurrence rate was 14.5%, which is similar to that noted in patients without gastrectomy history [14]. Risk factors of recurrent CBD stones in normal gastrointestinal anatomy patients were evaluated in previous studies [15,16,17]. The risk factors were revealed as GB stones, the use of mechanical lithotripsy, dilatation of CBD, periampullary diverticulum, distal CBD angulation ≤ 135–145°, and distal CBD stricture. We applied these risk factors to patients following gastrectomy in this study. We noted that the CBD stone recurrence rate was higher in patients with periampullary diverticulum (univariate analysis, *p* = 0.039) and CBD stricture (multivariable analysis, HR, 3.823; 95%, CI: 1.118–13.080). Diverticulum may facilitate bacterial colonization and consequently increase the intraluminal pressure in the duodenum as well as affect the function of the sphincter of Oddi, thereby increasing the risk of CBD stone recurrence [14]. Distal CBD stricture and the consequent narrowing of the lumen may impair bile flow, hinder access and the subsequent procedure for ERCP, and cause incomplete stone removal, thereby increasing the recurrence rate [18].

Regarding the procedure types and methods, our findings indicate a higher recurrence rate when CBD stones were removed using ERCP. The altered anatomy could be attributed to the difficulty in achieving complete stone removal. A more challenging endoscopic approach is needed in patients who underwent gastrectomy compared with those without gastrectomy history. Conversely, CBD stone removal through PTBD was not affected by changes in the gastroduodenal anatomy in patients who underwent gastrectomy. However, if the intubation of an endoscope is possible at the level of the ampulla of the duodenum, the following procedures of stone removal are not much different between ERCP and PTBD. The common treatments for the removal of CBD stones are using basket (and/or retrieval balloon catheter in ERCP) or balloon sphincteroplasty (or sphincterotomy in ERCP) in ERCP and PTBD procedures. Furthermore, we observed that the balloons used in ERCP-treated patients were smaller (<12 mm diameter) than those used in PTBD-treated patients (26.8% vs. 57.8%, *p* = 0.002). This difference in balloon size may have contributed to impaired bile flow. In the same vein, our study observed a lower recurrence rate when a 12 mm or larger balloon (21.1% vs. 53.6%, *p* = 0.009) was used for dilatation, possibly indicating improved bile flow. Therefore, the difference in effectiveness between ERCP and PTBD for recurrent CBD stones in this study is mostly affected by the retrieval balloon size and not the ERCP or PTBD modality itself. However, this finding remains controversial as it has also been reported that a larger bile duct sphincter opening size may actually increase reflux and increase the risk of CBD stone recurrence [14,19]. Further prospective studies with a larger sample size are necessary.

PTBD procedure has some disadvantages, such as higher complication rates (e.g., bleeding, bile peritonitis, etc.), catheter insertion site pain, and risk of dislodgement. Hepatocutaneous tract maturation time is also needed for about 2 weeks [20]. Therefore, additional procedure is necessary in PTBD, although CBD stone was removed. In our study, the number of procedures for CBD stone removal was higher in PTBD than ERCP group (3.71 ± 1.23 vs. 1.39 ± 0.67 (mean ± SD) times, *p* < 0.001). In contrast, ERCP techniques have been improved with the development of various endoscopy types and accessories (e.g., pediatric colonoscope or balloon enteroscope for total gastrectomy, cap-assisted upper gastrointestinal endoscope for subtotal gastrectomy Billroth II) to overcome the hurdles of altered anatomy in patients who underwent gastrectomy [8,21]. These advancements can reduce the difference in choledocholithiasis recurrence rate between ERCP and PTBD.

This study had some limitations. First, this study had a retrospective design; provided that patients who receive treatment for CBD stones do not undergo follow-up imaging, including computed tomography, unless their symptoms recur, it is possible that the recurrence rate was underestimated, considering that asymptomatic patients with recurrence would have been missed. Second, the sample size was small, and the complete follow-up period varied widely among patients. Third, the characteristics of CBD stones were mostly unknown because the CBD stones removed through PTBD could not be accessed in this study. To determine a more accurate recurrence rate and identify risk factors [20], prospective studies with larger sample sizes and regular follow-up imaging are warranted.

## 5. Conclusions

The incidence of CBD stones in patients who underwent gastrectomy is similar to that of the general population. ERCP, CBD stricture, and no use of balloon sphincteroplasty are potential risk factors for recurrent CBD stones following gastrectomy. When considering PTBD disadvantages, we recommend an ERCP procedure with active use of balloon sphincteroplasty to decrease the recurrent CBD stones.

## Figures and Tables

**Table 1:** Baseline demographic and clinical characteristics of the patients.

Characteristic	Patients Who Underwent CBD Stone Removal Following Gastrectomy (*N* = 131)
Sex, male, *n* (%)	100 (76.3)
Age, mean ± SD (years)	72.0 ± 9.4
Charlson comorbidity index score, mean ± SD, (points)	3.4 ± 1.9
Gastrectomy type, *n* (%)	
Subtotal gastrectomy with Billroth I	24 (18.3)
Subtotal gastrectomy with Billroth II	59 (45.0)
Total gastrectomy with Roux-en-Y	48 (36.6)
Treatment modality for CBD stones	
ERCP, *n* (%)	41 (31.3)
PTBD, *n* (%)	90 (68.7)
Duration between gastrectomy and CBD stone removal, mean ± SD (days)	183.7 ± 133.3
CBD stone, numbers, mean ± SD	1.7 ± 1.5
CBD stone size, mean ± SD (mm)	9.5 ± 4.4
CBD stone type, *n* (%)	
Brown	28 (21.4)
Black	3 (2.3)
Sludge	1 (0.8)
Unknown	99 (75.6)
Follow-up duration, mean ± SD (months)	31.5 ± 28.5

CBD, common bile duct; SD, standard deviation; ERCP, endoscopic retrograde cholangiopancreatography; PTBD, percutaneous transhepatic biliary drainage.

**Table 2:** Comparison of characteristics between the recurrent and non-recurrent groups.

	Recurrent Group (*N* = 19)	Non-Recurrent Group (*N* = 112)	*p*-Value
Sex, male, *n* (%)	15 (78.9)	85 (75.9)	0.772
Age (years)	73.0 ± 9.2	71.8 ± 9.5	0.616
ERCP stone removal, *n* (%)	10 (52.6)	31 (27.7)	0.030
Balloon sphincteroplasty, *n* (%)	8 (42.1)	84 (75.0)	0.004
Balloons larger than 12 mm, *n* (%)	4 (21.1)	60 (53.6)	0.009
Cholecystectomy, *n* (%)	5 (26.3)	28 (25.0)	1.000
GB stone/sludge, *n* (%)	11 (57.9)	67 (59.8)	0.874
Distal CBD angulation ≤ 135°, *n* (%)	14 (73.7)	88 (78.6)	0.765
CBD stricture, *n* (%)	4 (21.1)	8 (7.1)	0.074
CBD diameter, mean ± SD (mm)	13.5 ± 3.9	11.9 ± 3.6	0.089
Periampullary diverticulum, *n* (%)	5/11 (45.5)	5/36 (13.9)	0.039

SD, standard deviation; GB, gallbladder; CBD, common bile duct.

**Table 3:** Comparison of characteristics of subgroups divided by treatment modality.

	ERCP (*N* = 41)	PTBD (*N* = 90)	*p*-Value
Sex, male, *n* (%)	31 (75.6)	69 (76.7)	0.895
Age (years)	71.7 ± 9.7	72.1 ± 9.4	0.801
Charlson comorbidity index score, mean ± SD (points)	3.5 ± 2.0	3.3 ± 1.8	0.736
Gastrectomy type, *n* (%)			<0.001
Subtotal gastrectomy with Billroth I	20 (48.8)	4 (4.4)	
Subtotal gastrectomy with Billroth II	21 (51.2)	38 (42.2)	
Total gastrectomy with Roux-en-Y		48 (53.3)	
CBD stone recurrence, *n* (%)	10 (24.4)	9 (10.0)	0.030
Balloon sphincteroplasty, larger than 12 mm, *n* (%)	11 (26.8)	53 (58.9)	0.001
Balloon sphincteroplasty, *n* (%)	24 (58.5)	68 (75.6)	0.048

ERCP, endoscopic retrograde cholangiopancreatography; PTBD, percutaneous transhepatic biliary drainage; SD, standard deviation.

**Table 4:** Multivariable analysis of recurrent choledocholithiasis.

Variable	HR	95% CI	*p*-Value
PTBD	0.278	0.078–0.791	0.016
CBD stricture	3.823	1.118–13.080	0.033
Balloon sphincteroplasty	0.207	0.072–0.599	0.004

HR, hazard ratio; CI, confidence interval; PTBD, percutaneous transhepatic biliary drainage; CBD, common bile duct.

## Data Availability

The data presented in this study are available on request from the authors.

## References

[1] Figueiredo J.C., Haiman C., Porcel J., Buxbaum J., Stram D., Tambe N., Cozen W., Wilkens L., Le Marchand L., Setiawan V.W. (2017). Sex and ethnic/racial-specific risk factors for gallbladder disease. BMC Gastroenterol..

[2] Hedjoudje A., Cheurfa C., Et Talby M., Levy P., Prat F., Piton G. (2023). Outcomes and predictors of delayed endoscopic biliary drainage for severe acute cholangitis due to choledocholithiasis in an intensive care unit. Dig. Liver Dis..

[3] Liang C.-M., Chiu Y.-C., Lu L.-S., Wu C.-K., Sou F.-M., Chiu S.-M., Lee Y.-C., Huang P.-Y., Chuah S.-K., Kuo C.-M. (2022). Early and Direct Endoscopic Stone Removal in the Moderate Grade of Acute Cholangitis with Choledocholithiasis Was Safe and Effective: A Prospective Study. Life.

[4] Nakamura K., Ogoshi K., Makuuchi H. (2005). Clinicopathological study of cholelithiasis following gastric cancer surgery. Eur. Surg. Res..

[5] Kim S.Y., Bang W.J., Lim H., Lim M.S., Kim M., Choi H.G. (2019). Increased risk of gallstones after gastrectomy: A longitudinal follow-up study using a national sample cohort in Korea. Medicine.

[6] Tan Z., Xie P., Qian H., Yao X. (2019). Clinical analysis of prophylactic cholecystectomy during gastrectomy for gastric cancer patients: A retrospective study of 1753 patients. BMC Surg..

[7] Bencini L., Marchet A., Alfieri S., Rosa F., Verlato G., Marrelli D., Roviello F., Pacelli F., Cristadoro L., Italian Research Group for Gastric Cancer (GIRCG) (2019). The Cholegas trial: Long-term results of prophylactic cholecystectomy during gastrectomy for cancer-a randomized-controlled trial. Gastric Cancer.

[8] Yang M.J., Kim J.H., Hwang J.C., Yoo B.M., Park S.W., Kwon C.I., Jeong S. (2022). Mechanistic loop resolution strategy for short-type single-balloon enteroscopy-assisted endoscopic retrograde cholangiopancreatography in patients with Roux-en-Y reconstruction after gastrectomy (with video). Surg. Endosc..

[9] Kobayashi T., Hisanaga M., Kanehiro H., Yamada Y., Ko S., Nakajima Y. (2005). Analysis of risk factors for the development of gallstones after gastrectomy. Br. J. Surg..

[10] Zhang M., Zhang J., Sun X., Xu J., Zhu J., Yuan W., Yan Q. (2018). Clinical analysis of treatment strategies to cholecystocholedocholithiasis patients with previous subtotal or total gastrectomy: A retrospective cohort study. BMC Surg..

[11] Everhart J.E., Khare M., Hill M., Maurer K.R. (1999). Prevalence and ethnic differences in gallbladder disease in the United States. Gastroenterology.

[12] Zeng Q., He Y., Qiang D.C., Wu L.X. (2012). Prevalence and epidemiological pattern of gallstones in urban residents in China. Eur. J. Gastroenterol. Hepatol..

[13] Paik K.-H., Lee J.-C., Kim H.W., Kang J., Lee Y.S., Hwang J.-H., Ahn S.H., Park D.J., Kim H.-H., Kim J. (2016). Risk Factors for Gallstone Formation in Resected Gastric Cancer Patients. Medicine.

[14] Kato S., Chinen K., Shinoura S., Kikuchi K. (2017). Predictors for bile duct stone recurrence after endoscopic extraction for naive major duodenal papilla: A cohort study. PLoS ONE.

[15] Keizman D., Shalom M.I., Konikoff F.M. (2006). An angulated common bile duct predisposes to recurrent symptomatic bile duct stones after endoscopic stone extraction. Surg. Endosc..

[16] Costamagna G., Tringali A., Shah S.K., Mutignani M., Zuccala G., Perri V. (2002). Long-term follow-up of patients after endoscopic sphincterotomy for choledocholithiasis, and risk factors for recurrence. Endoscopy.

[17] Oh C.H., Dong S.H. (2015). Recent Advances in the Management of Recurrent Bile Duct Stones. Korean J. Gastroenterol..

[18] Nzenza T.C., Al-Habbal Y., Guerra G.R., Manolas S., Yong T., McQuillan T. (2018). Recurrent common bile duct stones as a late complication of endoscopic sphincterotomy. BMC Gastroenterol..

[19] Tsai T.J., Lin C.K., Lai K.H., Chan H.H., Wang E.M., Tsai W.L., Cheng J.S., Yu H.C., Chen W.C., Hsu P.I. (2018). Does preserved sphincter of Oddi function prevent common bile duct stones recurrence in patients after endoscopic papillary balloon dilation?. J. Chin. Med. Assoc..

[20] Hatjidakis A.A., Karampekios S., Prassopoulos P., Xynos E., Raissaki M., Vasilakis S.I., Gourtsoyiannis N.C. (1998). Maturation of the tract after percutaneous cholecystostomy with regard to the access route. Cardiovasc. Intervent. Radiol..

[21] Park T.Y., Kang J.S., Song T.J., Lee S.S., Lee H., Choi J.S., Kim H.J., Jang J.W. (2016). Outcomes of ERCP in Billroth II gastrectomy patients. Gastrointest. Endosc..

